# Downregulation of Complement C3 and C3aR Expression in Subcutaneous Adipose Tissue in Obese Women

**DOI:** 10.1371/journal.pone.0095478

**Published:** 2014-04-17

**Authors:** Abhishek Gupta, Reza Rezvani, Marc Lapointe, Pegah Poursharifi, Picard Marceau, Sunita Tiwari, Andre Tchernof, Katherine Cianflone

**Affiliations:** 1 Centre de Recherche Institut Universitaire de Cardiologie & Pneumologie de Québec, Université Laval, Québec, Canada; 2 Department of Physiology, King George’s Medical University, Lucknow, Uttar Pradesh, India; University of Warwick – Medical School, United Kingdom

## Abstract

**Background:**

The central component of the complement system, C3, is associated with obesity, metabolic syndrome and cardiovascular disease however the underlying reasons are unknown. In the present study we evaluated gene expression of C3, the cleavage product C3a/C3adesArg and its cognate receptor C3aR in subcutaneous and omental adipose tissue in women.

**Methods:**

Women (n = 140, 21–69 years, BMI 19.5–79 kg/m^2^) were evaluated for anthropometric and blood parameters, and adipose tissue gene expression.

**Results:**

Subjects were separated into groups (n = 34–36) according to obesity: normal/overweight (≤30 kg/m^2^), obese I (≤45 kg/m^2^), obese II (≤51 kg/m^2^), and obese III (≤80 kg/m^2^). Overall, while omental expression remained unchanged, subcutaneous C3 and C3aR gene expression decreased with increasing adiposity (2-way ANOVA, p<0.01), with a concomitant decrease in SC/OM ratio (p<0.001). In subcutaneous adipose, both C3 and C3aR expression correlated with apoB, and apoA1 and inversely with waist circumference and blood pressure, while C3aR also correlated with glucose (p<0.05–0.0001). While omental C3aR expression did not correlate with any factor, omental C3 correlated with waist circumference, glucose and apoB (all p<0.05). Further, while plasma C3a/C3adesArg increased and adiponectin decreased with increasing BMI, both correlated (C3a negatively and adiponectin positively) with subcutaneous C3 and C3aR expression (p<0.05–0.001) or less).

**Conclusions:**

The obesity-induced down-regulation of complement C3 and C3aR which is specific to subcutaneous adipose tissue, coupled to the strong correlations with multiple anthropometric, plasma and adipokine variables support a potential role for complement in immunometabolism.

## Introduction

Obesity and metabolic dysfunction are now associated with a state of chronic low-grade inflammation and the importance of that association is still emerging [1]. In recent years, there have been an increasing number of studies focusing on complement and innate immune system proteins in relation to obesity, inflammation, and dyslipidemias [2], [3], [4]. Studies in humans have demonstrated that C3, the most abundant component of the complement cascade, and its mediator molecules are increased in obesity, metabolic syndrome and cardiovascular disease [2], [5], [6], [7], [8], [9], [10]. However, in spite of the strong associations of C3 with obesity and metabolic syndrome, the underlying mechanisms remain unexplained.

Complement C3, lies at the heart of all three major complement activation pathways and plays a major role in inflammation [3]. C3 activation via the classical, alternative or lectin pathways results in the formation of a C3 convertase, which cleaves C3 to generate C3a in the tissue micro-environment [2]. C3a is then converted to C3adesArg (also known as ASP; Acylation Stimulating Protein) with removal of the carboxy terminal arginine via carboxypeptidases B or N [2]. Circulating C3a (which is primarily in the form of C3adesArg due to the rapid and efficient action of the carboxypeptidases) has been shown to be associated with various immune disorders such as ischemia/reperfusion injury, rheumatoid arthritis, and sepsis [11], [12], [13], as well as with metabolic disorders including obesity, diabetes, hyperlipidemia and coronary heart disease [2].

C3a acts through a G_i_-coupled G protein-coupled receptor, C3a receptor (C3aR) that transduces potent anaphylatoxin activity, directly triggering degranulation of mast cells, inflammation, chemotaxis and activation of leukocytes, as well as increasing vascular permeability and inducing smooth muscle contraction [14], [15]. Concurrently, C3adesArg/ASP, acting via its receptor C5L2, has potent lipogenic activity, stimulating glucose uptake and triglyceride storage in cultured adipocytes [2], [16] while *in vivo* human studies demonstrate local adipose tissue production of C3adesArg which may contribute to adipose tissue triglyceride metabolism [17], [18]. C3aR and C5L2, as well as the related receptor C5aR, fall within the same receptor family [19], sharing similar ligands (C5a/C5adesArg, C3a/C3adesArg) [20] reviewed in Klos A 2009 [14].

Receptors C5L2 and C5aR are present in murine adipose tissue [21], [22], and mouse knockout models lead to disturbances in whole body energy, lipid and glucose metabolism as well as altered adipose tissue [23], [24], [25]. On the other hand, little is known of the role of C3aR in adipose tissue although its presence in mouse adipose tissue (both primary adipocytes and macrophages) [26] and murine 3T3 adipocytes [22] has been confirmed. A recent paper indicated that C3aR knockout mice were more protected from high-fat diet induced metabolic dysfunction with a transient resistance to diet-induced obesity, but, there is no human data available [26].

We hypothesized that C3aR expression, its ligand; circulating C3a, and gene expression of the precursor complement C3 might be regulated in adipose tissue in relation to obesity. In the present study we evaluated gene expression of complement C3 and C3aR in omental and subcutaneous adipose tissue and plasma C3a/C3adesArg levels in Caucasian women, and their association with anthropometric and biochemical variables over a wide range of degrees of obesity.

## Materials and Methods

### Ethics Statement

Research protocols were approved by the CRIUCPQ (Centre de Recherche de l’Institut Universitaire de Cardiologie et Pneumologie de Québec) and CHUL (Centre Hospitalier de l’Université Laval) institutional review boards. All the participants provided written informed consent prior to the enrollment.

### Study Subjects

Samples were obtained from (i) severely obese women who had undergone weight-loss surgery (biliopancreatic diversion with duodenal switch, BPD-DS) at the CRIUCPQ and (ii) healthy women who had undergone elective surgery at the Gynecology Unit, Laval University Medical Center. Subjects with severe obesity were recruited through our institution-approved tissue bank for the study of causes and consequences of obesity (http://www.criucpq.ulaval.ca/index.php/en/tissue-bank). Selection criteria for bariatric surgery by the IUCPQ included excess body mass index (BMI), the presence of co-morbidities and a history of prior weight loss attempts. For the current study, women who had undergone bariatric surgery within a 3-year period (2007–2010) were evaluated for participation. Women who had previously undergone bariatric surgery were excluded. Additionally, subjects recruited met the following eligibility criteria for entry into the study: women aged between 21–69 years old, non-diabetic, not taking medication for dyslipidemia, had no other recognized co-morbidities, had not previously undergone ovariectomy, and availability of matched blood and adipose tissue samples. Individuals were classified into weight categories based on their BMI. In addition to the normal/overweight (N/Ow) category, defined as a BMI of less than or equal to 30 kg/m^2^, additional obese groups were defined as follows: BMI>30 to BMI≤45 (obese I), BMI>45 to BMI≤51 (obese II), and BMI>51 to BMI≤80 (obese III). Research protocols were approved by the CRIUCPQ and CHUL institutional review boards. All the participants provided written informed consent prior to the enrollment. Samples of subcutaneous (SC) and omental (OM) adipose tissue were excised under general anesthesia. All laboratory analyses were completed before statistical analysis was performed.

### Anthropometric Measurements

Women were assessed at baseline. Anthropometric measurements including body weight, height, and waist circumference were measured the day before surgery. Height was measured using a stadiometer (SECA, 216 1814009, Brooklyn, NY, USA). Weight was evaluated by electrical bio-impedance balance (Tanita TBF-310, Tokyo, Japan) following a 12-hour fast. BMI was calculated as weight (kg)/height (m^2^). Waist circumference (in cm) was measured at the narrowest point between the lowest rib and the iliac crest. Blood pressure was measured in the supine position on the right arm after rest (for at least 15 min). Medical history was collected for diabetes, hypertension, coronary artery disease and dyslipidemia as well as the corresponding pharmacological therapy. The information provided by the patient was confirmed by consulting clinical files.

### Blood Collection and Biochemical Assessment

Blood samples were collected after an overnight fast (12 h), and samples were immediately centrifuged (1000×g) for 10 min at 4°C. The plasma obtained was transferred to a fresh tube and frozen in aliquots at −80°C until further analysis. Assays were measured in the hospital clinical biochemistry laboratory using standard methodology for plasma cholesterol, triglyceride, HDL-cholesterol and glucose using colorimetric enzymatic kits (cholesterol, triglyceride and HDL-C: Roche Diagnostics Indianapolis, IN, USA; glucose: Wako Chemicals, Richmond, VA, USA). LDL-cholesterol was calculated using the Friedewald formula [27]. In the research laboratory; apolipoprotein (apo) B and A1 levels were measured by immunoturbidimetric method (Roche Diagnostics Integra 800 system). Plasma adiponectin was measured using an RIA kit (Millipore, USA). Plasma C3a/C3adesArg level was measured using an in-house modified ELISA method as previously published [28]. Note, antibodies used for this assay (and other commercial assays) cannot differentiate between C3a and C3adesArg, and it is primarily the C3adesArg form that is present in circulation.

Metabolic syndrome was defined according to the US National Cholesterol Education Program Adult Treatment Panel III guidelines and modified as recommended in the latest American Heart Association/National Heart, Lung, and Blood Institute Scientific Statement [29] by adopting a lower cutoff for fasting glucose (5.6 mmol/L). Metabolic syndrome was defined as having 3 of the following metabolic risk factors: (1) central obesity (waist circumference >88 cm in women), (2) hypertriglyceridemia (fasting triglycerides >1.69 mmol/L, (3) low HDL cholesterol (fasting HDL<1.29 mmol/L in women), (4) glucose intolerance (fasting glucose >5.6 mmol/L, and (5) hypertension (sitting blood pressure 130/85 mm Hg obtained as a mean of two readings taken after resting for at least 10 minutes.

### RNA Extraction and Real Time qPCR Analysis

Adipose tissue (SC and OM) samples were obtained at time of surgery and were immediately frozen in liquid nitrogen and kept at −80°C until used. All samples (maximum 100 mg) were homogenized in Qiazol (Qiagen Inc, Mississauga, ON, Canada) reagent. Total RNA was extracted from the homogenate according to RNeasy Plus Universal Mini Kit (Qiagen) manufacturer’s instructions. From the total amount, 0.1 µg of purified RNA was retro-transcribed to cDNA using QuantiTec Reverse Transcription Kit (Qiagen Inc., Mississauga, ON, Canada) for a final volume of 20 µL. Genomic DNA contamination was eliminated by DNase treatment included in QuantiTec Reverse Transcription Kit. For real-time PCR evaluation of gene expression, 1 µl of cDNA was used for each reaction. RT^2^ SYBR Green qPCR Master Mix (Qiagen Inc., Mississauga, ON, Canada) was used and a 3-step PCR was performed using CFX9 Real-Time PCR Detection System (Bio-Rad Laboratories, Mississauga, ON, Canada), using the following protocol: an initial denaturation step at 10 min at 95°C, 39 cycles of 95°C for 15 sec, 55°C for 40 sec, 72°C for 30 sec was followed by final extension step of 95°C for 10 sec. Real time RT-PCR was performed to quantitate human C3 and C3aR relative to glyceraldehyde-3-phosphate dehydrogenase (GAPDH); as a housekeeping gene. The sequences of the primers used were: C3 (Hs_C3_2_SG, NM_000064, QuantiTect primer assay, Qiagen, Inc., Mississauga, ON, Canada), C3aR1-Right; 5′-AGCAGAGAAAGACGCCATTG-3′, C3aR1-Left; 5′-ACTGTGGCTAAGTGTGGGGA-3′, and GAPDH-Right; 5′- AATGAAGGGGTCATTGATGG-3′, GAPDH-Left; 5′- AAGGTGAAGGTCGGAGTCAA-3′ (Alpha-DNA, Montreal, Canada). For data analysis the ΔΔCt method was used, as performed with Bio-Rad CFX manager software version 1.5 (Bio-Rad laboratories, Mississauga, ON, Canada).

### Statistical Analysis

All data are presented as mean ± standard error of the mean (sem). Normality of data was evaluated, and non-normally distributed data was log transformed prior to comparative analysis. Unpaired Student’s t-test, one-way ANOVA with linear trend or individual post-hoc tests, or 2-way ANOVA were used to evaluate differences for anthropometric, biochemical and gene expression parameters between different levels of obesity (N/Ow and Obese I, II and III groups) or between subcutaneous and omental adipose tissue (as indicated accordingly). Correlation analysis was evaluated by Pearson correlation test. Graph Pad Prism (San Diego, CA, USA) software program version 5.0 was used for graph and statistical analysis. P values <0.05 were considered to be statistically significant.

## Results

### Anthropometric and Biochemical Characteristics in Caucasian Women and Expression of C3 and C3aR in Subcutaneous and Omental Adipose Tissues

**Figure 1:**
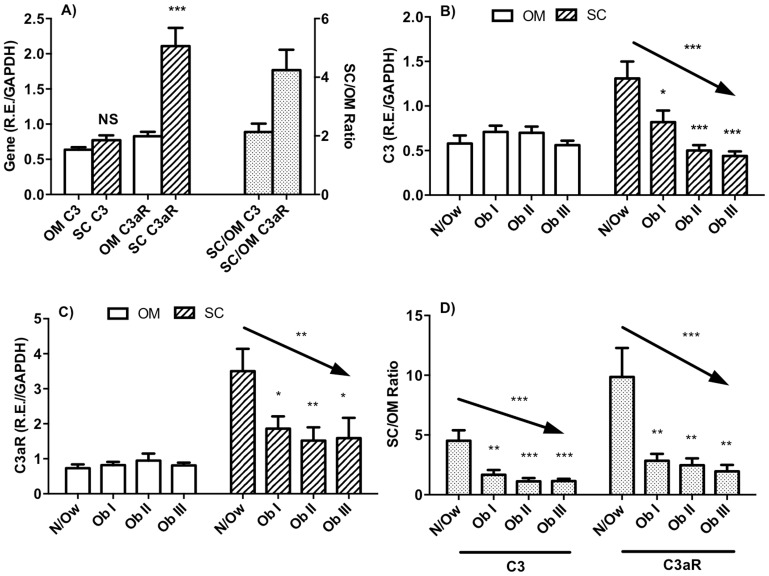
Expression levels of the target mRNAs in adipose tissue altogether and in normal/overweight and obese groups. Gene expression was determined by RT-qPCR and is presented as relative expression (R.E.) relative to housekeeping gene GAPDH. A) Expression of genes C3 and C3aR in OM and SC and ratio of SC to OM adipose tissue, B) and C) C3 and C3aR adipose tissue expression in N/Ow group and increasing obesity groups in OM and SC adipose tissue, and D) SC to OM adipose tissue expression level ratio of C3 and C3aR in N/Ow and various grades of obesity groups. Data is expressed as mean±SEM where *p<0.05, **p<0.01 and ***p<0.0001 and pNS indicates not significant.

Altogether 140 women ranging from 21 to 69 years (mean age 42.5±0.8), with BMI from 19.5 to 78.9 kg/m^2^ (mean BMI 43.0±1.1) were evaluated. The baseline anthropometric and biochemical characteristics are shown in Table 1. Expression of C3 and C3aR for all women in subcutaneous (SC) and omental (OM) tissues are shown in Figure 1A. While there was no overall difference in expression between OM and SC for C3, by contrast the levels of C3aR were much higher in SC (C3∶0.64±0.03 vs 0.77±0.07; p = NS and C3aR: 0.83±0.06 vs 2.11±0.26; p<0.0001, respectively). In addition, the ratio of SC relative to OM adipose tissue expression was much greater for C3aR than C3 (Figure 1A).

**Table 1:** Characteristics of anthropometric and biochemical variables in women.

Baseline	Mean ± SEM	Range
Age (years)	42.5±0.8	21.1–69.0
Height (cm)	161±1.0	135.0–178.0
Weight (kg)	112±3.0	48.0–201.4
BMI (kg/m^2^)	43.0±1.1	19.5–78.9
Waist circumference (cm)	127±2.5	71.5–184.0
Systolic BP (mm Hg)	130±1.4	85.0–186.0
Diastolic BP (mm Hg)	80.0±1.2	29.0–113.0
Glucose (mmol/l)	5.62±0.08	3.70–8.30
Total Cholesterol (mmol/l)	4.94±0.08	3.09–7.25
HDL-Cholesterol (mmol/l)	1.39±0.03	0.61–2.31
LDL-Cholesterol (mmol/l)	2.84±0.06	0.94–4.90
Triglyceride (mmol/l)	1.58±0.07	0.57–4.46
Apolipoprotein B (g/l)	0.89±0.03	0.27–2.27
Apolipoprotein A1 (g/l)	1.07±0.04	0.06–1.95
ApoB/Non HDL-C	0.25±0.01	0.08–0.62
LDL-C/ApoB	3.69±0.14	1.17–9.12

Range is given as minimum to maximum values.

Apo: apolipoprotein, BMI: body mass index, BP: blood pressure.

### General Characteristics of Anthropometric and Biochemical Variables in Women Grouped by Obesity

Women were separated into 4 groups according to their degree of obesity based on BMI: i) Normal/Overweight (N/Ow; >19 to <30 kg/m^2^), ii) Obese I (>30 to <45 kg/m^2^), iii) Obese II (>45 to <51 kg/m^2^), and iv) Obese III (>51 to <79 kg/m^2^) as presented in Table 2. One way ANOVA analysis demonstrated significant differences for anthropometric and biochemical variables including metabolic syndrome parameters waist circumference, systolic and diastolic blood pressure, triglyceride and HDL-C as well as related factors TC/HDL-C, LDL-C/ApoB, ApoA1, as well as age, with specific group differences indicated (Table 2). As many of these parameters are used in evaluation of the presence/absence of metabolic syndrome, this was evaluated in the four groups. As shown in Table 2, the N/Ow group had relatively few subjects with metabolic syndrome (8 of 35, 23%), while there was significantly more in the three obese groups (66%, 79% and 72%), although all to the same extent.

**Table 2:** Characteristics of Normal/Overweight and Obese groups.

Variables	N/Ow	Obese I	Obese II	Obese III	ANOVA
Age (years)	46.7±0.7	42.8±1.5*	38.9±1.9***	41.5±1.9*	0.006
Height (cm)	162±1	164±1*	161±1	160±1	NS
Weight (kg)	65±1.4	106±2.4***	125±1.6***	151±3.6***	<0.0001
BMI (kg/m^2^)	25.0±0.5	39.3±0.8***	48.3±0.3***	59.1±1.2***	<0.0001
WC# (cm)	87.4±1.4	125.3±2.7***	141.7±1.5***	156.8±2.7***	<0.0001
SBP# (mm Hg)	114±2.3	131±2.1***	138±2.7***	138±2.2***	<0.0001
DBP# (mm Hg)	68±2.1	80±1.9***	84±1.9***	86±2.1***	<0.0001
Glucose# (mmol/l)	5.76±0.12	5.43±0.15	5.38±0.14*	5.91±0.19	NS
Total Chol (mmol/l)	4.94±0.15	4.93±0.18	4.94±0.16	4.95±0.12	NS
HDL-Chol# (mmol/l)	1.56±0.06	1.35±0.06*	1.31±0.06**	1.35±0.05**	0.006
LDL-Chol (mmol/l)	2.80±0.12	2.83±0.16	2.86±0.13	2.85±0.11	NS
Triglyceride# (mmol/l)	1.26±0.09	1.64±0.14*	1.78±0.16**	1.65±0.12**	0.026
ApoB (g/l)	0.94±0.04	0.88±0.08	0.86±0.07	0.87±0.07	NS
ApoA1 (g/l)	1.45±0.04	1.13±0.06***	0.82±0.07***	0.91±0.07***	<0.0001
LDL-C/ApoB	3.01±0.05	3.74±0.28*	4.09±0.37**	3.89±0.29**	0.016
ApoB/Non HDL-C	0.28±0.003	0.25±0.02	0.25±0.02	0.24±0.02*	NS
Metabolic Syndrome	(8/27)	(23/12)	(27/7)	(26/10)	<0.0001
(Yes/No), %	23%	66%	79%	72%	

Values are given as average ± SEM for each group (34–36 per group) where N/Ow represents the normal/overweight group, with three obese groups (I, II and III of increasing obesity). Data were analyzed by ANOVA (or Chi^2^ for metabolic syndrome distribution), followed by post-hoc test for comparison vs normal/overweight group where *p<0.05, **p<0.01 and ***p<0.0001 and p NS indicates not significant. ApoB and ApoA1, apolipoprotein B and A1; BMI, body mass index; Chol, cholesterol; DBP, diastolic blood pressure; SBP, systolic blood pressure; WC, waist circumference.

# Parameters used for diagnosis of metabolic syndrome.

### C3 and C3aR Expression Levels in Subcutaneous and Omental Adipose Tissue Relative to Body Size in Women

For both C3 and C3aR, obesity levels did not affect gene expression in OM tissue (Figure 1B and C). However in subcutaneous tissue, C3 mRNA was significantly decreased with increasing BMI, with significant differences as compared to the N/Ow group (p<0.0001, ANOVA linear trend, Figure 1B). As shown in Figure 1C, similar results were obtained for C3aR expression in subcutaneous tissue, with decreasing levels with increasing obesity, and significant differences in the obese groups as compared to N/Ow group (p = 0.008, ANOVA linear trend). Further, when analyzed individually as the ratio of SC to OM adipose tissue (paired samples, Figure 1D), there was a significant decrease in both complement C3 and C3aR receptor gene expression overall, with significant decrease in all obese groups as compared to N/Ow group (both p<0.0001, ANOVA linear trend).

### Correlation of C3 and C3aR Gene Expression with Clinical and Metabolic Parameters in Adipose Tissue

**Figure 2:**
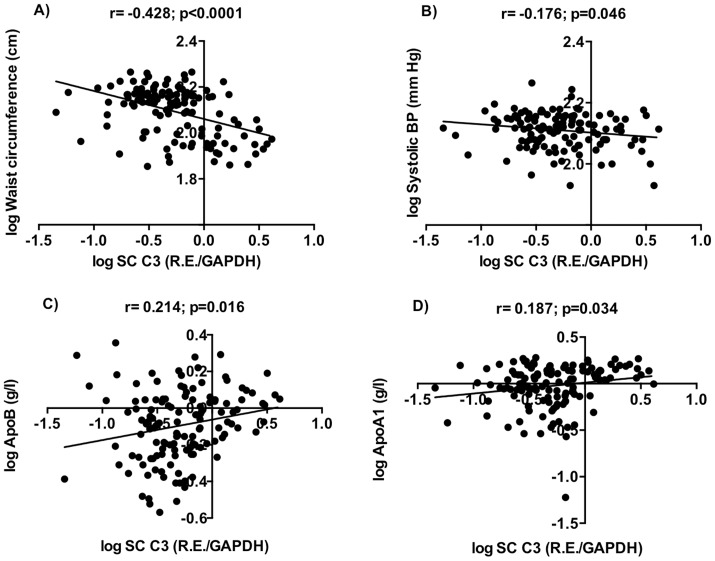
Relationship between subcutaneous C3 mRNA levels, anthropometric and plasma variables. Correlations are shown between C3 and A) waist circumference (WC), B) Systolic blood pressure (Systolic BP), C) Apolipoprotein B (ApoB), and D) apolipoprotein A1 (ApoA1) in subcutaneous adipose tissue. For correlation, results are presented as Pearson correlation by linear regression.

Significant correlations of complement C3 with clinical and metabolic parameters were noted, particularly in subcutaneous adipose tissue, including negative correlation with waist circumference (r = −0.428; p<0.0001, Figure 2A) and systolic blood pressure (r = −0.176; p = 0.046, Figure 2B) as well as positive correlations with ApoB and ApoA1 (r = 0.214; p = 0.016 and r = 0.187; p = 0.034, Figure 2C and D respectively). Subcutaneous C3 also correlated negatively with BMI and diastolic blood pressure (r = −0.449; p<0.0001 and r = −0.212; p = 0.016, respectively) and positively with ApoB/NonHDL-C (r = 0.176; p = 0.048).

For omental adipose tissue, C3 expression correlated positively with waist circumference r = 0.177; p = 0.042) and negatively with glucose and ApoB (r = −0.175; p = 0.043 and r = −0.170; p = 0.05, respectively). Further, the ratio of SC relative to OM adipose tissue (SC/OM) for C3 correlated negatively with BMI, waist circumference, systolic and diastolic blood pressure and positively with fasting plasma glucose, ApoB and ApoB/NonHDL-C (data not shown).

**Figure 3:**
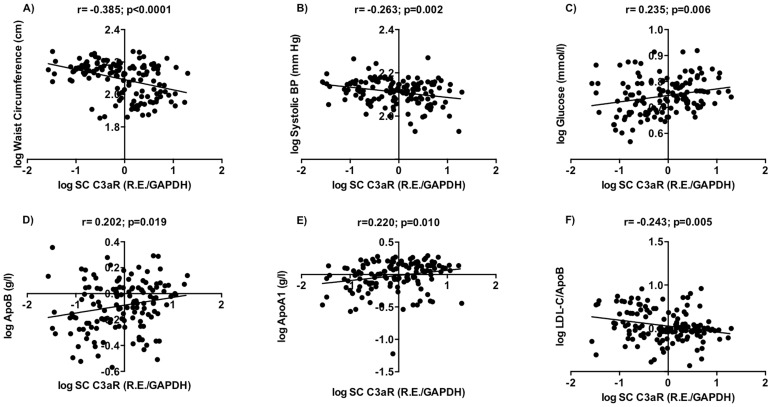
Relationship between subcutaneous C3aR mRNA levels, anthropometric and plasma variables. Correlations are shown between C3aR and A) Waist circumference, B) Systolic blood pressure (Systolic BP), and plasma parameters C) Glucose, D) Apolipoprotein B (ApoB), E) Apolipoprotein A1 (ApoA1), and F) LDL-C/ApoB in subcutaneous adipose tissue. For correlation, results are presented as Pearson analysis by linear regression.

With C3aR gene expression, in subcutaneous adipose tissue there were negative correlations with waist circumference (r = −0.385; p<0.0001, Figure 3A) and systolic blood pressure (r = 0.263; p = 0.002, Figure 3B). Plasma parameters that correlated included positive correlations with plasma glucose (r = 0.235; p = 0.006, Figure 3C), apolipoproteins ApoB and ApoA1 (r = 0.202; p = 0.019 and r = 0.220; p = 0.010, Figure 3D and E respectively) and inversely correlated with LDL-C/ApoB (r = −0.243; p = 0.006, Figure 3F). In addition, subcutaneous C3aR negatively correlated with diastolic blood pressure but positively correlated with ApoB/NonHDL-C (r = −0.239; p = 0.005 and r = 0.231; p = 0.007, respectively). Strikingly, in omental adipose tissue there were no significant correlations of C3aR with any parameter.

### C3a/C3adesArg and Adiponectin Levels in Normal/Overweight and Obese Groups and their Correlation with C3 and C3aR Expression Levels in Subcutaneous Adipose Tissue

**Figure 4:**
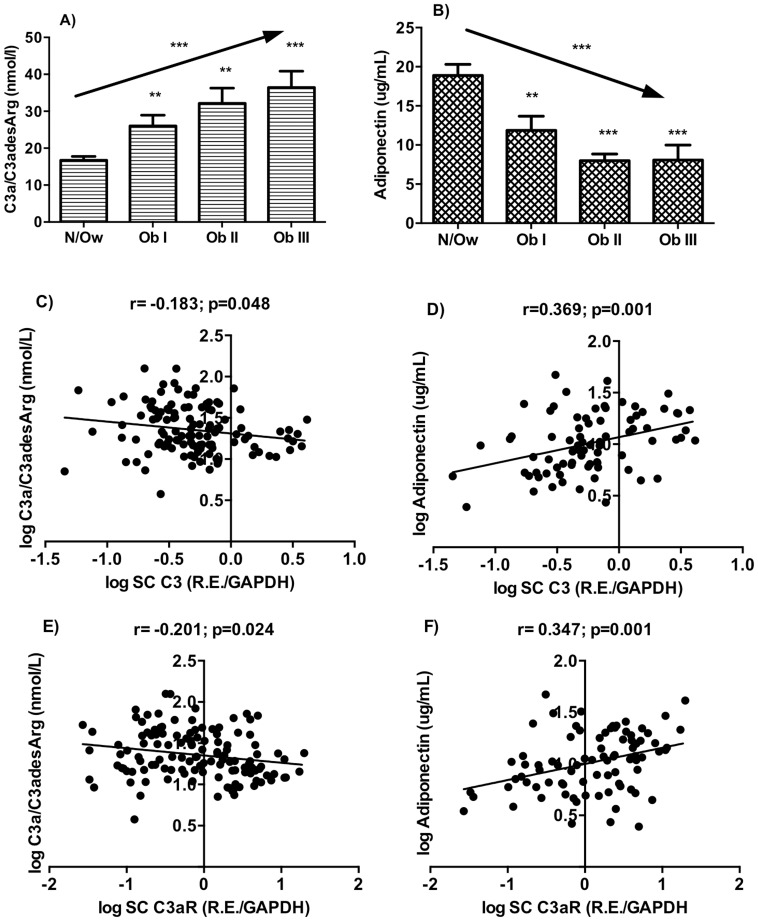
Plasma levels of C3a/C3adesArg and adiponectin and their relationship with subcutaneous C3 and C3aR mRNA gene expression levels. A) Plasma C3a/C3adesArg and B) adiponectin in Normal/overweight (N/Ow) group and increasing obesity groups. Plasma C3a/C3adesArg correlates negatively with C3 (C) and C3aR (E) gene expression whereas adiponectin correlates positively with C3 (D) and C3aR (F) in subcutaneous adipose tissue. Data are expressed as mean±SEM where **p<0.01 and ***p<0.0001. For correlation, results are presented as Pearson analysis by linear regression.

C3a, the ligand for C3aR, which is present in plasma primarily as C3adesArg, as well as adiponectin, an adipokine implicated in metabolic function, were also evaluated. A stepwise increase in plasma C3a/C3adesArg levels and a graded decrease in adiponectin levels were found with increasing obesity levels (Figure 4A and B, both p<0.0001, ANOVA linear trend) with significant differences between the individual obesity groups and N/Ow group.

There was a significant negative correlation between subcutaneous C3 mRNA levels and plasma C3a(desArg) levels (r = −0.183; p = 0.048, Figure 4C) whereas a positive correlation was found with plasma adiponectin levels (r = 0.369; p = 0.001, Figure 4D). Similarly, a significant negative correlation was found between subcutaneous C3aR expression levels and plasma C3a/C3adesArg levels (r = −0.201; p = 0.024, Figure 4E) whereas a positive correlation was found with adiponectin levels (r = 0.347; p = 0.001, Figure 4F). Significant positive correlations of plasma C3a/C3adesArg levels with BMI, waist circumference, systolic blood pressure, triglyceride levels, ApoB levels and ApoB/NonHDL-C and negative correlations with LDL-C/ApoB ratio were also observed (data not shown).

## Discussion

While many studies have linked serum complement C3 and its cleavage products to type 2 diabetes, metabolic syndrome and cardiovascular diseases, and C3 levels are related to body fat, the underlying mechanisms explaining this association are still unknown [3], [2]. Few studies have directly evaluated complement C3 in adipose tissue in humans, and, to date, no studies have evaluated the relevance of C3aR expression in human adipose tissue. In the present study, the salient and novel findings are that with increased BMI, the mRNA expression levels of C3 and C3aR in women were decreased in subcutaneous adipose tissue but unchanged in omental adipose tissue. Further, we show for the first time that C3aR expression is higher in the subcutaneous adipose depot versus omental. In addition, the SC/OM ratio of C3 and C3aR expression decreases with obesity suggesting a down-regulation in human SC adipose tissue. Our study also demonstrated a strong association of both complement C3 and C3aR expression levels in SC adipose tissue with adiposity, lipoprotein metabolic markers, plasma C3a/C3adesArg and adiponectin levels, with little association in OM adipose tissue.

Circulating complement C3 is increased in metabolic disorders and typically correlates with adiposity development, and metabolic syndrome parameters such as plasma triglyceride levels (reviewed recently in [3], [30], [31]. However the mechanism is unknown, and while C3 is produced by the liver, it is also synthesized by adipose tissue [32], [33], and specifically by human adipocytes in a differentiation dependent manner [34]. However, there are very few studies that have examined C3 expression in SC vs OM adipose tissues to distinguish between depot-specific differences, and in several there is a lack of tissue availability from non-obese subjects [35], [33]. The results are conflicting with both no difference between SC and OM [36], or higher OM C3 expression versus SC [37], [38]. However, these studies suffer from limited sample size (9–32 subjects) and a mixed population of men and women. Here, using a large sample size (n = 140) we observe higher expression of C3 in SC vs OM in normal/overweight subjects, but comparable expression over a range in obesity (as demonstrated by the SC/OM ratio).

It is interesting that SC adipose tissue C3 mRNA expression decreases with increasing adiposity given that plasma C3 levels are usually positively associated with obesity, metabolic dysfunction, insulin resistance, cardiovascular disease and type 2 diabetes [39], [9], [7], [2] while partial lipodystrophy is associated with hypocomplementemia [40]. This reduction of C3 expression in SC is in agreement with what was previously demonstrated by Xia [36] for mRNA expression and by Insenser M [41] for protein expression. We speculate that, in spite of a down-regulation of SC C3 gene expression, the large increase in SC adipose tissue mass in obesity might counteract this effect and contribute to the increased circulating C3 levels. Further, there are contributions from other sources, such as the increased mass of OM adipose tissue and alternate sources including liver, a major site of C3 production [32] which exceeds adipose expression [33].

Similarly, obesity/metabolic dysfunction is associated not only with increased circulating C3, but also C3 cleavage products, such as C3a/C3adesArg [2]. The source of these cleavage products includes direct production in adipocytes through interaction of C3 with alternative pathway components factor B and adipsin/complement factor D [34]. In the present study, circulating C3a/C3adesArg increases with obesity, consistent with other studies (review [2]). However the circulating C3a/C3adesArg levels do not positively correlate with either SC or OM C3 mRNA expression levels. This lack of correlation between precursor-product has also been noted in studies evaluating circulating C3 protein and C3a/C3adesArg [2], [42] and suggests that it is not the level of C3 (either tissue mRNA or circulating protein) that is the determining factor in C3a/C3adesArg production, but rather regulation of the enzymatic cleavage process. In fact, circulating factors such as dietary chylomicrons and factor H have been shown to contribute to C3a/C3adesArg production *in vitro* [12] **,** in cultured adipocytes [34] and in human studies [17], [18].

In contrast to the supporting data available on C3, there is no human data available supporting a role for C3aR in adipose tissue as C3aR, a G protein-coupled receptor [43], has been primarily evaluated from an immunological perspective [15]. However, two recent rodent studies have identified an adipose tissue link. In rodents, C3aR is expressed in significant amounts in cultured 3T3 adipocytes [22] as well as in adipose tissue adipocytes and macrophages, in addition to liver and muscle [26]. Using various tools including a mouse knockout model, integrated genomics and targeted antagonist approaches, C3aR has been implicated in omental fat mass, diet-induced obesity, and metabolic dysfunction [22], [26], [44]. First, in a study using C3aR knockout mice, C3aR was proposed to be a determinant of metabolic dysfunction, where C3aRKO mice were transiently protected from the metabolic effects of high fat diet/obesity-induced insulin resistance and adipose tissue inflammation [26]. Second, Lim et al., (2013) [22] proposed a preventive role of C3aR antagonists in high carbohydrate diet-induced obesity, metabolic dysregulation, and adipose tissue macrophage infiltration in rats. The present study is the first study on human adipose tissue: we observed higher C3aR mRNA levels in SC vs. OM adipose tissue with decreased C3aR expression levels in SC adipose tissue with increasing obesity, which also suggests a role for C3aR in adipose tissue function. As there is a significant increase in plasma C3a/C3adesArg levels, this inverse correlation (more ligand C3a coupled to less receptor C3aR) suggests a potential ligand-mediated down-regulation of the C3aR gene, indicative of the development of a “resistant” state.

Our study demonstrates that, with increasing BMI, C3 and C3aR expression were significantly decreased in SC adipose tissue but unchanged in OM adipose tissue. These relative changes are best appreciated with the SC/OM ratio, with 5- and 10-fold ratios for C3 and C3aR, respectively, in the non-obese group, with rapid reduction at any level of obesity. This ties into the observations that C3aR KO mice are transiently resistant to weight gain, protected from high-fat diet-induced metabolic dysfunction and have less macrophage infiltration into adipose tissue [26], while C3KO mice are leaner, resistant to diet-induced obesity, with greater energy expenditure [45], [46], [47] and mice with increased C3 activation leading to excess C3a/C3adesArg (as seen in CD55KO and IL6KO mice) have increased adiposity [48], [49]. The study results are striking in that the majority of correlations with various anthropomorphic and circulating factors are found in SC but not OM, and are similar for C3 and C3aR with positive correlations for both genes with adiponectin, apoB and apoA1, and inverse correlations for both with BMI, waist circumference, blood pressure indices and C3adesArg. We speculate that the joint C3–C3aR down-regulation might be a protective mechanism set into motion with the development of excess adiposity, and an attempt at protection against obesity-related complications. Acutely, C3a increases both C3aR and C3 [50], [51], however chronic stimulation with high circulating levels, as in obesity, may lead to negative feedback down-regulation, although the precise mechanism by which this is achieved is unknown.

The limitations of our study should be noted. While the sample size is large, with subjects presenting a wide range of BMI, all of the samples are from women. Further, this represents a cross-sectional study, limiting the potential for cause-and-effect interpretations.

In summary, our study shows that (i) complement component C3 and C3aR gene expression are specific to adipose tissue depot (subcutaneous vs. omental), (ii) are influenced by body size and level of adiposity in SC only, and (iii) C3 and C3aR expression are strongly associated with anthropometric parameters, lipoprotein markers, and the adipokines C3a/C3adesArg and adiponectin in women.
